# Internet use, social engagement and health literacy decline during ageing in a longitudinal cohort of older English adults

**DOI:** 10.1136/jech-2014-204733

**Published:** 2014-11-26

**Authors:** Lindsay C Kobayashi, Jane Wardle, Christian von Wagner

**Affiliations:** Department of Epidemiology and Public Health, University College London, London, UK

**Keywords:** AGEING, COGNITION, LONGITUDINAL STUDIES, SOCIAL INEQUALITIES, Social activities

## Abstract

**Background:**

Health literacy skills tend to decline during ageing, which is often attributed to age-related cognitive decline. Whether health literacy skills may be influenced by technological and social factors during ageing is unknown.

**Methods:**

We investigated whether internet use and social engagement protect against health literacy decline during ageing, independent of cognitive decline. We used prospective data from 4368 men and women aged ≥52 years in the English Longitudinal Study of Ageing from 2004 to 2011. Health literacy was measured at baseline (2004–2005) and at follow-up (2010–2011) using a reading comprehension test of a fictitious medicine label. The influences of consistent internet use and engagement in each of the civic, leisure and cultural activities on health literacy decline over the follow-up were estimated.

**Results:**

After adjusting for cognitive decline and other covariates, consistent internet use (1379/4368; 32%) was protectively associated with health literacy decline (OR=0.77; 95% CI 0.60 to 0.99), as was consistent engagement in cultural activities (1715/4368; 39%; OR=0.73; 95% CI 0.56 to 0.93). As the number of activities engaged in increased, the likelihood of health literacy decline steadily decreased (p_trend_<0.0001), with OR=0.51 (95% CI 0.33 to 0.79) for engaging in all four of the internet use and civic, leisure and cultural activities versus none.

**Conclusions:**

Internet use and social engagement, particularly in cultural activities (eg, attending the cinema, art galleries, museums and the theatre), may help older adults to maintain health literacy during ageing. Support for older adults to maintain socially engaged lives and to access the internet should help promote the maintenance of functional literacy skills during ageing.

## Introduction

Ageing involves rising challenges for health and well-being. During ageing, adults often have increased contact with the healthcare system as risk increases for several chronic diseases and services such as certain types of cancer screening become available. However, age-related cognitive changes and health literacy decline may compromise the ability to navigate the healthcare system and use health information.^1–3^ Health literacy is closely related to literacy, and is defined by the Institute of Medicine as the degree to which individuals have the capacity to obtain, process and understand basic health information and services needed to make appropriate health decisions.^4^ Among older adults, low health literacy is associated with poor self-care of chronic diseases, excess use of emergency care services, low use of preventive health services and increased risk of overall mortality.^5–8^

A growing body of literature indicates that the most salient explanatory factor in ageing-related health literacy decline is cognitive function.^9–13^ In the absence of cognitive impairment, the ‘fluid’ cognitive abilities that are involved in active learning (eg, working and prospective memory; inductive reasoning) together with ‘crystallised’ cognitive abilities (eg, vocabulary; generalised knowledge) explain over 70% of the association between health literacy skills and performance on health tasks.^10^ Fluid cognitive ability has been shown to decline in a non-pathological manner during ageing beginning in mid-adulthood,^14–16^ and also to mostly explain low health literacy among older adults.^17^ ^18^ However, the influences of technological and social factors that involve active learning and cognitive stimulation on health literacy during ageing have never been investigated in a longitudinal manner.

Cross-sectional research has found that adults with lower health literacy are less likely to use the internet than those with adequate health literacy.^19^ ^20^ Furthermore, a randomised online eHealth intervention focused around the learning of new health information has been shown to improve the performance of adults diverse by age and culture on health literacy assessments.^21^ Internet use has also been longitudinally associated with the maintenance of cognitive function during ageing.^22–24^ Randomised evidence shows that trained use of tablet computers, involving use of the internet in practical applications, improves executive function over social and non-intellectually stimulating activities among older American adults.^25^

With respect to social engagement, a body of longitudinal research with long-term follow-up shows that a diverse range of social activities including physical activity, intellectual game-playing, membership in religious and other social groups and participation in cultural activities all protect against several measures of cognitive decline during ageing.^26–31^ However, in a shorter 3-month randomised trial of socially and cognitively stimulating activities, cognitive function was not improved in a social engagement group of older adults (activities such as field trips that were novel but did not involve active learning), whereas it was in a ‘receptive engagement’ group (active learning of novel skills).^32^ Evidently, this body of knowledge is still evolving, although it appears that cognitively-stimulating social activities may help maintain cognitive function during ageing. This relationship may extend to health literacy.

We hypothesise that internet use and engagement in intellectually-stimulating social activities may have positive effects on the maintenance of health literacy skills during ageing. The objective of this study is to investigate the roles of internet use and social engagement (in civic, leisure and cultural activities) in health literacy decline during ageing among a population-based sample of English adults aged ≥52 years in the English Longitudinal Study of Ageing (ELSA).

## Methods

### English Longitudinal Study of Ageing

ELSA is a longitudinal cohort study representative of the English population aged ≥50 years, established in 2002 as a stratified random sample of a private household.^33^ Data are collected biennially through computer-assisted in-person interviews and nurse-conducted health assessments are performed every 4 years. ELSA was approved by the London Multicentre Research Ethics Committee (MREC/01/2/91) and informed consent was obtained from all participants. The present analysis uses data from ELSA participants aged ≥52 years in waves 2 (2004–2005), 3 (2006–2007), 4 (2008–2009) and 5 (2010–2011) of the ELSA data collection. Eligible participants were ‘core’ ELSA members recruited in the original data collection wave (2002–2003), who participated in all data collection waves with non-proxy interviews (n=5133).

### Study measures

#### Health literacy

Health literacy was measured at waves 2 (baseline) and 5 (follow-up) of ELSA during the in-person study interview. Participants were shown a fictitious medicine label similar to that found on a bottle of aspirin and asked four reading comprehension questions by the interviewer (see online supplementary material 1). The measure was developed by the Organisation for Economic Co-operation and Development (OECD) and Statistics Canada for the Adult Literacy & Life Skills Survey^34^ to reflect the goal-based, clinically-relevant health task of understanding and interpreting instructions on a medicine label. Health literacy decline was defined as decreasing in score by ≥1 point between waves 2 and 5.^6^

#### Internet use

Data on internet use were collected in a self-completion questionnaire that ELSA participants completed in addition to the in-person interview. Internet use was assessed at each wave using a checklist item, ‘I use the internet and/or email’. ‘Never users’ were those who did not tick the item in any wave, ‘Intermittent users’ inconsistently ticked the item across waves 2–5, and ‘Consistent users’ ticked the item in all waves.

#### Social engagement

Social engagement is conceptualised in this analysis using an index of ‘social detachment’ from ELSA.^33^ The index includes a range of civic, leisure and cultural activities that would use diverse cognitive abilities including those involved in active learning (table 1). A crude social network measure (ie, having friends, children or other immediate family and being in contact with them at least once per week) was included in the index but not used in the present study, due to the low variability in response to the variable and a lack of empirical evidence for the association between this variable and cognitive stimulation. Data were collected in the self-completion questionnaire, where participants ticked off the statements relating to them in each wave. Participants were categorised as being engaged or not engaged in each domain at each wave. Across waves, social engagement was described as being ‘Consistent’, Intermittent’ or ‘None’ for each domain.

**Table 1:** Civic, leisure and cultural activities classified in the English Longitudinal Study of Ageing

Civic activities	Current member of a political party; ▸ trade union or environmental group; ▸ tenants’ or neighbourhood group or neighbourhood watch; ▸ church or religious group; ▸ charitable association; And did volunteer work in the past year
Leisure activities	Current membership in a ▸ social club; ▸ sports club; ▸ gym or exercise class; ▸ other organisation, club or society
Cultural activities	In the past year, attending a ▸ cinema; ▸ art gallery or museum; ▸ theatre, concert or opera performance

#### Covariates

Sociodemographic and health-related covariates considered as potential confounders were: age at wave 2 of the data collection, sex, ethnicity (white; non-white), educational attainment (degree or equivalent; up to degree level; no qualification), net non-pension wealth quintile (stratified at age 65 to account for retirement), having a limiting long-standing illness (yes; no), and experiencing a limitation in any instrumental activity of daily living (IADL) over the follow-up period (yes; no). Cognitive covariates were: baseline memory (score out of 27 on the memory index, consisting of time orientation, immediate and delayed recall, and prospective memory), baseline executive function (score out of infinity on the executive function index, consisting of verbal fluency and mental processing speed), memory decline, and executive function decline (yes or no for a decline of >1 point on the index for each wave between waves 2 and 5). 35

### Study sample

At wave 2, 5024/5133 (97.9%) eligible participants completed the health literacy assessment. Reasons for non-completion of the health literacy assessment were sight problems (n=35), health problems (n=9) and refusals without a reason or a non-codeable reason given (n=65). At wave 5, 4853/5133 (94.5%) eligible participants completed the health literacy assessment and therefore had a follow-up health literacy measure. Reasons for non-completion at wave 5 were: sight difficulties (n=85), health problems (n=33), reading problems (n=25), other problems such as anxiety, illness or other mental impairment (n=29), or refusal without a reason given (n=77). Overall, 4837/5133 (94.2%) participants had complete health literacy data, 4710/5133 (91.8%) had complete cognitive function data and 4859/5133 (94.7%) had net non-pension wealth data. When all missing data were accounted for, the final sample size was 4368. The effective sample size for the multivariable-adjusted models was 4365, as two participants were missing data on ethnicity and one on education.

### Statistical analysis

Sample characteristics were analysed bivariately against health literacy decline using the χ^2^ test for categorical variables and Student's t test for continuous variables. The proportions of internet users and those engaged in each social domain were calculated for each data collection wave. Multivariable logistic regression was used to estimate ORs and 95% CIs for the associations between internet use and engagement in each of the civic, leisure and cultural activities (four main effects) and health literacy decline over the 6-year follow-up. Three model sets were run: model set 1 was for the associations between each main effect and health literacy decline, adjusted for sociodemographic and health-related covariates; model 2 adjusted for all main effects simultaneously in addition to covariates; model 3 additionally included baseline cognitive function and cognitive decline variables. A secondary analysis investigated the additive effect of maintaining engagement in one, two, three or all four of the internet use and civic, leisure and cultural activities over the follow-up. To avoid the baseline adjustment bias, baseline health literacy was not adjusted for.^36^ We ran a multiple imputation analysis to account for missing health literacy and cognitive function data (see online supplementary material 2). All analyses were conducted using StataSE V.13.1 (StataCorp, College Station, Texas, USA).

## Results

At baseline, 3187/4368 participants (73%) had adequate health literacy (table 2). Over the 6-year follow-up, 814/4368 participants (19%) declined by one or more point in the health literacy score, while 791/4368 (18%) improved by one or more points. The proportion of adults who declined in the health literacy score increased with age (p<0.0001), while improvement was non-differential by age (p=0.42). Being older, non-white, in a lower wealth quintile, having no educational qualifications, and experiencing at least one IADL limitation over the follow-up were associated with health literacy decline (table 2). Lower memory and executive function scores at baseline were associated with health literacy decline, as was experiencing cognitive decline over the follow-up (table 2).

**Table 2:** Characteristics of study participants by health literacy decline, the English Longitudinal Study of Ageing, 2004–2011 (n=4368)

	Health literacy decline		
	Yes (n=814; 19%)	No (n=3554; 81%)	p Value
Age			<0.0001
Mean (SD)	66.97 (8.99)	64.03 (8.09)	
Sex			0.37
Male	369 (19%)	1549 (81%)	
Female	445 (18%)	2005 (82%)	
Ethnicity			0.02
White	797 (18%)	3513 (82%)	
Non-white	17 (30%)	39 (70%)	
Educational attainment			<0.0001
Degree or equivalent	149 (14%)	925 (86%)	
Up to degree level	330 (17%)	1656 (83%)	
No qualification	334 (26%)	973 (74%)	
Non non-pension wealth quintile			<0.0001
1 (poorest)	155 (24%)	501 (76%)	
2	154 (19%)	653 (81%)	
3	180 (20%)	703 (80%)	
4	155 (16%)	808 (84%)	
5 (richest)	170 (16%)	889 (84%)	
Limiting long-standing illness			0.17
No	366 (18%)	1692 (82%)	
Yes	448 (19%)	1862 (81%)	
IADL limitation over study follow-up			<0.0001
No	542 (17%)	2649 (83%)	
Yes	272 (23%)	905 (77%)	
Baseline health literacy			<0.0001
Adequate	656 (21%)	2531 (79%)	
Limited	158 (13%)	1023 (87%)	
Baseline memory (range: 4–27)			<0.0001
Mean (SD)	15.45 (3.83)	16.93 (3.62)	
Baseline executive function (range: 5–23)			<0.0001
Mean (SD)	12.80 (3.12)	13.92 (3.04)	
Memory decline			<0.0001
No	488 (17%)	2429 (83%)	
Yes	379 (22%)	1313 (78%)	
Executive function decline			<0.0001
No	521 (17%)	2616 (83%)	
Yes	346 (24%)	1126 (76%)	

IADL, instrumental activity of daily living.

Across the data collection waves, 1755/4368 participants (40%) reported never using the internet or email and 1234/4368 (32%) consistently reported their use. Across waves, 1539/4368 participants (35%) were consistently engaged in civic activities, 1373/4368 (31%) in leisure activities and 1715/4368 (39%) in cultural activities. Participation across civic, leisure and cultural activities was significantly but modestly correlated with Spearman's r, ranging from 0.31 to 0.37 (p<0.0001 for all). Among those who experienced health literacy decline, internet use and engagement in all three social domains were lower at each wave than in those who did not decline (p<0.0001 for all waves).

In logistic regression adjusted for sociodemographic and health-related covariates, consistent internet use was protectively associated with health literacy decline (OR=0.60; 95% CI 0.49 to 0.76 vs never use), as was consistent engagement in civic and cultural activities (table 3). When all four main effects were mutually adjusted for in the same model, the association with civic activities was attenuated to the null. When cognitive variables were entered into the model, the associations with internet use and cultural engagement were somewhat attenuated, but remained statistically significant. The OR for consistently engaging in any one of the internet use, civic, leisure or cultural activities versus no engagement was 0.93 (95% CI 0.76 to 1.14), compared to 0.81 (95% CI 0.63 to 1.02) for engaging in any two activities, 0.70 (95% CI 0.53 to 0.94) for engaging in any three activities, and 0.51 (95% CI 0.33 to 0.79) for engaging in all four activities, a significant linear trend in effects (p_trend_<0.0001; table 4).

**Table 3:** Associations between internet use, social engagement and health literacy decline, the English Longitudinal Study of Ageing, 2004–2011 (n=4368)

	Health literacy decline							
Activities	Yes (n=814; 19%)	No (n=3554; 81%)	Model 1*	95% CI	Model 2†	95% CI	Model 3‡	95% CI
Internet use								
Never	435 (25%)	1320 (75%)	1.00		1.00		1.00	
Intermittent	218 (18%)	1016 (82%)	0.85	(0.69 to 1.03)	0.86	(0.70 to 1.05)	0.92	(0.75 to 1.13)
Consistent	161 (12%)	1218 (88%)	0.60	(0.49 to 0.76)	0.68	(0.54 to 0.87)	0.77	(0.60 to 0.99)
Civic activities								
None	263 (23%)	876 (77%)	1.00		1.00		1.00	
Intermittent	317 (19%)	1373 (81%)	0.83	(0.69 to 1.01)	0.87	(0.71 to 1.05)	0.85	(0.70 to 1.04)
Consistent	234 (15%)	1305 (85%)	0.72	(0.58 to 0.89)	0.82	(0.65 to 1.02)	0.84	(0.67 to 1.06)
Leisure activities								
None	246 (21%)	905 (79%)	1.00		1.00		1.00	
Intermittent	360 (20%)	1484 (80%)	1.05	(0.87 to 1.27)	1.14	(0.94 to 1.39)	1.18	(0.97 to 1.44)
Consistent	208 (15%)	1165 (85%)	0.85	(0.68 to 1.06)	1.05	(0.83 to 1.33)	1.12	(0.88 to 1.42)
Cultural activities								
None	227 (26%)	637 (74%)	1.00		1.00		1.00	
Intermittent	368 (21%)	1421 (79%)	0.88	(0.72 to 1.07)	0.90	(0.73 to 1.10)	0.92	(0.75 to 1.14)
Consistent	219 (13%)	1496 (87%)	0.60	(0.47 to 0.75)	0.67	(0.53 to 0.87)	0.73	(0.56 to 0.93)

*Adjusted for age, sex, ethnicity, educational attainment, net non-pension wealth, having a limiting long-standing illness, experiencing an IADL limitation.

†Model 1+internet use and engagement in each of the civic, leisure and cultural activities.

‡Model 2+baseline executive function, baseline memory, executive function decline and memory decline.

IADL, instrumental activity of daily living.

**Table 4:** The additive effects of internet use and social engagement on health literacy decline, the English Longitudinal Study of Ageing, 2004–2011 (n=4368)

	OR*	95% CI
Per additional activity	0.87	(0.81 to 0.94)
Number of activities engaged in†		
None	1.00	
One	0.93	(0.76 to 1.14)
Two	0.81	(0.63 to 1.02)
Three	0.70	(0.53 to 0.94)
Four	0.51	(0.33 to 0.79)

*Adjusted for age, sex, ethnicity, educational attainment, net non-pension wealth, having a limiting long-standing illness, experiencing an IADL limitation, baseline executive function, baseline memory, executive function decline and memory decline.

†The four activities referred to are internet use and each of civic, leisure, and cultural activities.

IADL, instrumental activity of daily living.

The multiple imputation analysis yielded similar results to the complete-case analysis (see online supplementary material 2).

## Discussion

In this longitudinal cohort of English adults aged 52 years and above, consistent internet use and engagement in cultural activities including attending the theatre, cinema, art galleries, museums, concerts or opera at least once a year were individually associated with ageing-related health literacy decline in a protective manner. As the number of activities engaged in increased, the protective association with health literacy decline increased in magnitude. When all four of the internet use, civic, leisure and cultural activities were consistently engaged in, the protective association was the strongest. These relationships were independent of cognitive function and decline.

### Strengths and weaknesses of this study

Our health literacy measure was developed, validated, and used by the OECD for an international adult literacy survey.^34^ Although individual validation metrics were unavailable for the measure, performance on it is associated with participation in cancer screening^6^ and all-cause mortality,^7^ showing its predictive capability for health outcomes. A limitation is that the clinically relevant task of understanding a medicine label is not representative of the diverse situations in which health literacy applies. The health literacy field is plagued by this problem, whereby objective instruments that comprehensively measure health literacy according to its multiple and sometimes conflicting definitions are not yet developed.^37^

Our four-point scale displayed a ceiling effect common to measures of health literacy.^38^ ^39^ The narrow scale range also resulted in few participants declining by more than one point over the follow-up: of those who declined (814/4368), 585 declined by one point, 170 by two points, 50 by three points, and 9 by four points. Owing to these small numbers, we could not discriminate between these magnitudes of decline as outcomes. Similar yet more comprehensive instruments also use health artefacts,^38^ but are less practical for incorporation in long, interview-based studies like ELSA. Improvement in the health literacy score over time was non-differential by age, indicating that decline is an age-related phenomenon and not due to random response error.

As with any population-based study, two main limitations are non-response bias and attrition bias. Older, non-white and less-educationally qualified adults were under-represented in wave 2 of ELSA (81.5% response rate) and were more also likely to drop out of the study between waves 2 and 5 (42% overall attrition rate among core members). These demographic factors were significantly associated with internet use, social engagement and health literacy decline. Therefore, we may have underestimated the magnitudes of the protective associations that we observed due to differential attrition. Our chance of a type I error may be inflated due to the multiple associations we tested. Missing data are unlikely to bias the results of this study, as the results were negligibly altered in the multiple imputation analysis.

Cognitive function was assessed in the face-to-face study interview using multiple validated measures of memory and executive function.^36^ However, we had no measure of reasoning, which is a predictor of health literacy.^10^ Therefore, our cognitive indices may have been less strongly associated with health literacy than those including reasoning. We were also unable to measure specific activities within each domain of social engagement (eg, specific types of volunteer work, educational classes and theatre shows). The cognitive demands of specific activities would vary, which could have caused a non-differential misclassification biasing of our results to the null. This important lack of information and the potential for reverse causality due to our longitudinal, but not necessarily causal, models limit the extent to which we and others can make hypotheses about the specific cognitive learning processes through which health literacy may be improved. However, our findings open several areas of inquiry regarding the mechanisms through which internet use and social engagement may influence health literacy skills.

### Comparison with other studies

The longitudinal relationships between internet use, social engagement and health literacy have never been investigated to the best of our knowledge, particularly never among an ageing sample. Our hypothesis regarding internet use and health literacy decline was supported, consistent with two previous cross-sectional studies.^19^ ^20^ It appears from our study that the relationship between internet use and health literacy decline is only partly explained by memory and executive function. Although we could not investigate these pathways, internet use may also promote health literacy skills through:

- Providing opportunities for health knowledge acquisition;
- Improving other cognitive functions such as reasoning;
- Providing social benefits through social media and networking sites;
- Training the specific navigational and digital skills required to use the internet.

Our hypothesis that intellectually-stimulating social engagement would be protective against health literacy decline was mostly supported. It may be that the categories in the leisure activity domain were too general to observe an association, and/or that they involved passive engagement, which has no effect on cognitive function.^32^ With respect to cultural activities, these would most likely engage several fluid cognitive abilities depending on the specific show or exhibit. Importantly, the association we observed was independent of wealth, education, having a limiting long-standing illness or experiencing IADL limitations over the follow-up, indicating that cultural engagement is not a proxy for socioeconomic circumstances or good physical capability.

Although additional studies with more comprehensive cognitive function measures are needed, we found that internet use and social engagement were associated with health literacy independently of memory and executive function measures. However, health literacy has recently been postulated to be little more than a marker of cognitive function.^11^ ^40^ Our findings suggest that even if this were the case, it would be premature to assume an overly deterministic view of health literacy. Ignoring health literacy, and by extension all literacy, as an innate or predetermined ability would be a disservice to all those who experience literacy-based barriers to health and well-being in society. This paper highlights the usefulness of putting health literacy in the context of both cognitive and social functions, particularly when trying to better understand changes to health literacy skills in later life.

## Conclusion

Our results indicate that internet use and social engagement may help older adults to maintain the functional literacy skills required to manage health. Individually, internet use and cultural engagement appeared to have beneficial associations. Together, all four factors appeared to act in an additive fashion, with the more the better for maintaining literacy skills. Further studies with additional cognitive, technological and social measures are needed for consideration alongside ours. The socially-embedded and intersecting problems of low internet use, low social engagement and health literacy decline are complex and will most likely require multimodal interventions to overcome. As an early longitudinal investigation in this area, our study should bring about several new hypotheses about the social and cognitive processes that influence the dynamics of literacy at older ages, and how they may be modified.

**What is already known on this subject**

- Cross-sectional research consistently shows an association between older age and low health literacy, which is often attributed to cognitive ageing. There is no longitudinal evidence for modifiable technological or social influences on health literacy skills during ageing.

**What this study adds**

- Internet use and engagement in various social activities, in particular cultural activities, appear to help older adults maintain the literacy skills required to self-manage health. These factors appeared to act in an additive fashion, with the more the better for maintaining literacy skills. Results indicate that health literacy skills are fluid over time, that loss of literacy skills during ageing is not inevitable, and that technological and social factors should be understood as influences on literacy skills.

## Supplementary Material

Web supplement

Web supplement
