# Supplementary material for: Internet use, social engagement and health literacy decline during ageing in a longitudinal cohort of older English adults
Source: J Epidemiol Community Health. 2014 Nov 26;69(3):278–83. doi: 10.1136/jech-2014-204733 (PMC4345520; doi:10.1136/jech-2014-204733)
Supplement: Web supplement [file jech-2014-204733-s1.pdf]

Supplemental Material 1: Health literacy assessment

MEDCO TABLET
INDICATIONS: Headaches, muscle pains, rheumatic pains, toothaches, earaches.

RELIEVES COMMON COLD SYMPTOMS

DOSAGE: ORAL. 1 or 2 tablets every 6 hours, preferably accompanied by food, for not longer than 7 days. Store in a cool, dry place.

CAUTION: Do not use for gastritis or peptic ulcer. Do not use if taking anticoagulant drugs. Do not use for serious liver illness or bronchial asthma. If taken in large doses and for an extended period, may cause harm to kidneys. Before using this medication for chicken pox or influenza in children, consult with a doctor about Reyes Syndrome, a rare but serious illness. During lactation and pregnancy, consult with a doctor before using this product, especially in the last trimester of pregnancy. If symptoms persist, or in the case of an accidental overdose, consult a doctor. Keep out of reach of children.

INGREDIENTS: Each tablet contains
500 mg acetylsalicylic acid.
Excipent c.b.p 1 tablet
Reg. No. 88246

Made in Canada by STERLING PRODUCTS. INC
1600 Industrial Blvd. Montreal, Quebec H9J 3P1

INSTRUCTIONS READ OUT BY THE INTERVIEWER
The final task in this section is about comprehension. This is a made-up medicine label and does not refer to a real medicine. It is often difficult to read and understand instructions on medicine labels. In a moment, I will ask you to read the card quietly to yourself. I will then ask you some questions about what it says. You do not have to memorise the card, as you will be able to look at it while answering the questions.

1) What is the maximum number of days you may take this medicine?
   (Correct answer 7. If responds with 'one week', interviewer may probe for number of days. Other answers incorrect.)

2) List three situations for which you should consult a doctor.
   (Respondent should mention at least three of the following: (Before giving medication to children with) chicken pox, (Before giving medication to children with) influenza,

Reyes syndrome, (During) lactation, (During) pregnancy, If symptoms persist, (Accidental) overdose. Incorrect answer: any other response.)

3) List one condition for which you might take the Medco tablet.
   (Correct if answered one of: Headaches, Muscle pains, Rheumatic pains, Toothache, Earache, Common cold. Other answers incorrect.)

4) List one condition for which you should not take the Medco tablet.
   (Correct if respondent mentions at least one of the following as conditions for which you should not take the tablet: Gastritis, Peptic ulcer, Serious liver illness, Bronchial asthma. Incorrect answer: any other response.)

Scoring: 1 point per complete correct response.
