# Supplementary material for: Internet use, social engagement and health literacy decline during ageing in a longitudinal cohort of older English adults
Source: J Epidemiol Community Health. 2014 Nov 26;69(3):278–83. doi: 10.1136/jech-2014-204733 (PMC4345520; doi:10.1136/jech-2014-204733)
Supplement: Web supplement [file jech-2014-204733-s2.pdf]

Supplementary Material 2: Methods and results for multiple imputation to account for missing data

In the core ELSA sample who participated in each of waves 2 through 5 with non-proxy interviews (n=5133), 2.1% (109/5133) did not complete the health literacy assessment at wave 2, 4.8% (246/5133) did not complete the health literacy assessment at wave 5, 0.3% (16/5133) did not complete the memory index at wave 2, 9.8% (108/5133) did not complete the executive function index at wave 2, 3.0% (152/5133) did not complete the memory index at wave 5, and 7.1% (364/5133) did not complete the executive function index at wave 5. Little's MCAR test indicated that the data were not missing completely at random ($\chi^2(4)=169.85$, $p<0.0001$ for the health literacy variables and $\chi^2(37)=238.52$, $p<0.0001$ for cognitive function variables). Data were not missing in a monotone fashion. Missing values were imputed using multivariate normal regression (an iterative Markov Chain Monte Carlo method) to create 20 imputed datasets. Absolute health literacy score at waves 2 and 5, absolute scores for memory and executive function at waves 2 and 5 were imputed from the model, which included baseline age, sex, ethnicity, educational attainment, internet use, engagement in each of civic, leisure, and cultural activities, and self-rated sight, as sight problems were a cause for non-completion of the health literacy assessment. The imputation model included 5129/5133 participants, as one was missing data on education and two on ethnicity. Health literacy decline and cognitive decline (memory and executive function) were specified as 'passive' variables in the multiple imputation dataset, meaning that their values are wholly dependent on the imputed variables; the Stata command 'mi passive: replace' was used to calculate their values from the imputed dataset. Note that net non-pension wealth, with 280/5133 (5.3%) observations missing, was non-imputable and not included in the imputation model, to little consequence. The Supplementary Table below shows the results for all logistic regression models using the imputed data.

Supplementary Material 2: Methods and results for multiple imputation to account for missing data

Supplementary Table 1. Multivariable logistic regression models with multiple imputation for missing values of health literacy and cognition (n=5129)

	ORs for health literacy decline (yes vs. no)					
	Model 1*	95% CI	Model 2†	95% CI	Model 3†‡	95% CI
Internet use						
Never	1.00		1.00		1.00	
Intermittent	0.55	(0.46, 0.65)	0.78	(0.65, 0.93)	0.85	(0.71, 1.03)
Consistent	0.41	(0.34, 0.50)	0.65	(0.52, 0.81)	0.76	(0.60, 0.96)
Engagement in:						
Civic activities						
None	1.00		1.00		1.00	
Intermittent	0.73	(0.62, 0.87)	0.86	(0.72, 1.03)	0.87	(0.73, 1.05)
Consistent	0.54	(0.45, 0.65)	0.80	(0.65, 0.98)	0.85	(0.69, 1.05)
Leisure activities						
None	1.00		1.00		1.00	
Intermittent	0.79	(0.67, 0.92)	1.05	(0.88, 1.25)	1.11	(0.92, 1.32)
Consistent	0.55	(0.46, 0.66)	0.94	(0.76, 1.16)	1.03	(0.83, 1.29)
Cultural activities						
None	1.00		1.00		1.00	
Intermittent	0.62	(0.53, 0.74)	0.82	(0.68, 0.98)	0.90	(0.74, 1.08)
Consistent	0.36	(0.30, 0.43)	0.64	(0.51, 0.80)	0.74	(0.58, 0.93)

*Unadjusted
†Adjusted for age, sex, ethnicity, educational attainment, internet use, having a limiting long-standing illness, experiencing any IADL limitation, and participation in each of civic activities, leisure activities, and cultural activities
‡Additionally adjusted for baseline executive function, baseline memory, executive function decline, and memory decline
